# Effects of Usnic Acid to Prevent Infections by Creating a Protective Barrier in an In Vitro Study

**DOI:** 10.3390/ijms24043695

**Published:** 2023-02-12

**Authors:** Rebecca Galla, Sara Ferrari, Sara Ruga, Beatrice Mantuano, Giorgia Rosso, Stelvio Tonello, Luigi Rosa, Piera Valenti, Francesca Uberti

**Affiliations:** 1 Laboratory of Physiology, Department of Translational Medicine, University of Piemonte Orientale, Via Solaroli 17, 28100 Novara, Italy; 2 Noivita Srls, Spin-Off UPO, Via Alfieri 3, 28100 Novara, Italy; 3 Laboratory of Immuno Rheumatology-Internal Medicine, Department of Translational Medicine, CAAD Center for Allergic and Autoimmune Disease, Via Solaroli 17, 28100 Novara, Italy; 4 Department of Public Health and Infectious Diseases, University of Rome La Sapienza, Piazzale Aldo Moro, 5, 00185 Rome, Italy

**Keywords:** usnic acid, nasal spray, mechanical barrier, virus protection

## Abstract

Nasal sprays are medical devices useful for preventing infection and the subsequent spread of airborne pathogens. The effectiveness of these devices depends on the activity of chosen compounds which can create a physical barrier against viral uptake as well as incorporate different substances with antiviral activity. Among antiviral compounds, UA, a dibenzofuran derived from lichens, has the mechanical ability to modify its structure by creating a branch capable of forming a protective barrier. The mechanical ability of UA to protect cells from virus infection was investigated by analyzing the branching capacity of UA, and then the protection mechanism in an in vitro model was also studied. As expected, UA at 37 °C was able to create a barrier confirming its ramification property. At the same time, UA was able to block the infection of Vero E6 and HNEpC cells by interfering with a biological interaction between cells and viruses as revealed also by the UA quantification. Therefore, UA can block virus activity through a mechanical barrier effect without altering the physiological nasal homeostasis. The findings of this research could be of great relevance in view of the growing alarm regarding the spread of airborne viral diseases.

## 1. Introduction

The nasal cavity is a primary site of the defense mechanism against airborne pathogens as it protects the respiratory tract by involving mucosal immunity in the airways [1]. Since this natural barrier is very efficient, several studies have turned interest on this defense mechanism to develop simple and safe interventions aimed at improving this physiological function to prevent respiratory infections such as influenza or COVID-19. Indeed, the epithelium of the airways of the nasal mucosa produces a quantity of mucus that traps the pathogens that are discharged from the cilia into the nasopharynx [1,2,3]. Mucociliary clearance and immune responses work together to protect the nasal epithelium from pathogens, but in some cases, they are not sufficient to prevent their entry into the airways. In this context, it could be important to improve the mechanical barrier by using an additional element which, combined with anti-infective drugs, could enhance this natural protective mechanism [3]. In this scenario, nasal sprays are a useful tool containing substances for preventing the subsequent spread of respiratory pathogens in sick individuals. In this regard, it is important to incorporate any molecule that may participate in the creation of a physical barrier capable of blocking the entry of the virus [4]. Several strategies have been tested to block airborne viruses, such as Carragelose^®^ nasal sprays, Nasaleze^®^ Cold & Flu blocker, and Vicks^®^ First Defense Cold Virus Blocker. Recently the same nasal sprays have been tested to be used as a prophylactic for the Sars-CoV-2 virus and its variants [1,2,3]. The effectiveness of these strategies depends on their ability to maintain effects locally without reaching other tissues to rule out cytotoxicity, as clearly indicated by the FDA draft guide for nasal sprays [5]. These passive barriers can be generated using different gelling agents such as carrageenan or chitosan or other ingredients that can prolong the retention time of the gelling agent on the nasal mucosa [4,6], also modulating the viscosity and droplet size [7].

In this context, of great importance is hydroxypropyl methylcellulose (HPMC) [8] which is a cellulose derivative, well known for the ability to create a “mucosal barrier” when used in nasal spray [9]. The clinical real-life benefits of HPMC in the nose depend on its efficiency gelling property to trap all particles present in the air [8]. Indeed, there are studies that explore the opportunity to add other ingredients such as antimicrobial drugs of natural origin to the gelling barrier [10]. There are natural substances with antiviral effects that could be an interesting starting point for developing a strategy to control the infectious diseases [11]. One of these is usnic acid (UA), a dibenzofuran derived from lichen [10]. UA exists in two enantiomeric forms, depending on the position of the methyl group at the chiral atom, which have no substantial differences in physical properties, but have significant differences in their biological and pharmacological activity [12]. UA has been extensively studied, but the results regarding its benefits in relation to the enantiomeric form and method of extraction are controversial [13]. For example, it is well known that (+)-usnic acid could induce weight loss in humans [14], unfortunately high UA consumption revealed hepatotoxic effects. However, UA could have other beneficial functions including antimicrobial, insecticide, anticholinergic, antioxidant, pro-oxidant, antigenotoxic, genotoxic, teratogenic, anti-inflammatory, analgesic and antipyretic, mutagenic, carcinogenic, and antitumor activity [13]. Moreover, most of the published data concern (+)-usnic acid, while, only a small number of reports refer to the activity of (+)- and (−)-usnic acid revealing identical antibacterial activity for both enantiomers whereas anti-viral activity is more dependent on (+)-enantiomer [12]. For this reason, UA is also used to inhibit virus replication [15] and it was identified in silico as one of the over 20,000 natural product that might be able to act against SARS-CoV-2 [10]. It should also be considered that one of the key properties of UA is based on its mechanical ability to modify its structure by creating a branch which, in contact with other UA molecules, is able to form a protective barrier [16] already tested against fungal hyphae [17] as well as against other infectious agents [18]. When its branching power due to its chemical form is combined with the polymeric capacity of HPMC, an improvement in its formula is achieved [19]. To achieve this, it is important to improve the solubility of the UA to prepare the nasal spray according to the regulatory deal on medical devices. In this context, it is known that the use of 2-hydroxypropyl-beta-cyclodextrin (*β*-CD) to facilitate the solubility of the compounds [20] prevents a UA toxic effect [21]. Moreover, to prevent the damage by inhibiting the radical oxygen species production (ROS), the addition of tocopherol and tocotrienol (named TPmix) to the final nasal spray formula has been proposed [22].

Taking these characteristics into consideration, it was possible to hypothesize and create a formulation suitable for use as a medical device. The purpose of this study, in fact, is to demonstrate that the nasal spray composed of UA + *β*-CD + HPMC + TPmix, called HEXEDRA+ as it is registered as a Class II Medical Device, is able to create a mechanical barrier to trap the virus. For this reason, the study aimed to investigate for the first time the ability of HEDEXRA+ to prevent infection of Vero E6 and nasal epithelial cells by VSV-based SARS-CoV-2 pseudovirus.

## 2. Results

### 2.1. Branching Capacity of Usnic Acid

**Figure 1:**
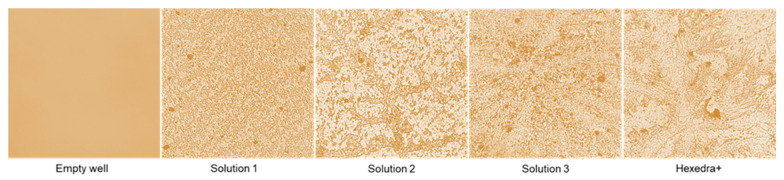
Crosslinking analysis by staining. The images reported are an example obtained from each treatment obtained at 40× magnification from four technical replicates.

One of the properties of UA reported in the literature is based on its mechanical ability to modify its structure by creating a branch that can form a protective barrier against several pathogens. As reported in Figure 1, this ability becomes visible at 3 h, confirming that the preparations can build the barrier when UA is placed at 37 °C for about 3 h and that this barrier remains anchored to the support. However, it is very delicate and can be easily removed when washed. As can be seen, the complete formulations that have been tested (Solution 1, 2, 3, and HEXEDRA+) create different crosslinkers with dissimilar porosity, probably due to the different composition (in addition to UA there is also the presence of TPmix in HEXEDRA +). Furthermore, the amount of the UA is also important to create the barrier. In fact, as revealed by Solution 2 and Solution 3, the balance between UA and HPMC is important to create more ramifications, and this improves the trapping property of the final formulation. Therefore, the preparation HEXEDRA+ not only possesses the branching capacity of UA but possesses a different conformation with a denser network and broad branches in places, making it suitable to be formulated as a medical device.

To confirm that UA could branch the bottom of the plate, forming a mechanical barrier, the presence of UA was evaluated analyzing the wash buffer and the bottom plate wash. As reported in Table 1, quantification of UA showed that UA was present in all formulations tested, remaining in both the wash buffer and in the bottom wash, demonstrating the ability of UA to remain anchored at the bottom of the plate and supporting its mechanical barrier activity.

### 2.2. ACE2 and TMPRSS2 Expression

Considering that the interaction between the Spike protein and angiotensin-converting enzyme 2 (ACE2) and transmembrane serine protease 2 (TMPRSS2) receptors is the main mechanism of entry of the virus into the cell, the expression of these receptors was evaluated in two cell types. The African green monkey kidney (Vero E6) cells have higher receptor expression, thus proving to be a suitable experimental model for virus infection and subsequent replication. Similarly, the human primary nasal cells (HNEpC) showed a suitable framework (Table 2).

### 2.3. Infection with VSV-Based Pseudovirus SARS-CoV-2 in Vero E6 Cells

**Figure 2:**
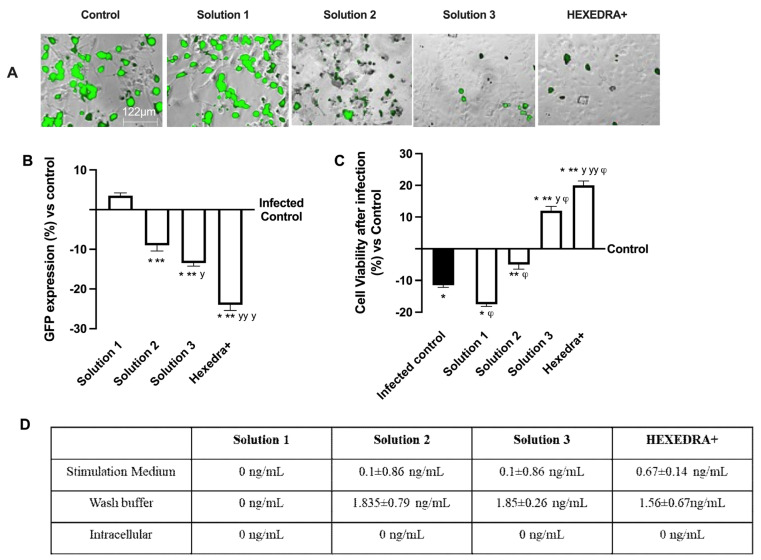
Analysis of infection of Vero E6 cells with VSV-based pseudovirus SARS-CoV-2 (MOI 0.1) pre-treated with the different UA-based formulations. In (**A**), the images reported were an example of each stimulation for GFP fluorescence expression obtained at 40× magnification by Paula (Leica DM1000); in (**B**) the analysis of GFP expression in cells obtained by the image analysis using Image J Software and the results were expressed as mean ± SD (%) compared to infected control cells (0 line) of four independent experiments. In (**C**), cell viability measured by MTT test after infection and expressed as mean ± SD (%) compared to uninfected control cells (0 line) obtained from four independent experiments. In (**D**) UA spectrophotometry quantification at 281 nm by Tecan (Infinite 200 Pro Mplex, Tecan, Milan, Italy) and data are expressed as ng/mL ± SD of four independent experiments. Solution 1 = 150 mcg/mL *β*-CD +HPMC; Solution 2 = 150 mcg/mL UA+ 150 mcg/mL *β*-CD + 1% HPMC; Solution 3 = 75 mcg/mL UA+ 150 mcg/mL *β*-CD + 1% HPMC; HEXEDRA+ = 75 mcg/mL UA+ 150 mcg/mL *β*-CD + 0.1% Tpmix + 1% HPMC. * *p* < 0.05 vs. Control; ** *p* < 0.05 vs. Solution 1; ^y^ *p* < 0.05 vs. Solution 2; ^yy^ *p* < 0.05 vs. Solution 3; ^φ^ *p* < 0.05 vs. Infected control.

The ability of the recombinant vesicular stomatitis virus (VSV)-based SARS-CoV-2 pseudovirus at a multiplicity of infection (MOI), 0.1 to infect Vero E6 cells, was verified, as well as the ability of UA to prevent infection. In particular, it is possible to observe in Figure 2A,B that the control is infected but with reduced vitality due to the virus infection which also determines a certain degree of cytotoxicity to favor the inflammatory state (about 10% compared to cells not infected, Figure 2C). Infection was revealed by the fluorescence rate observed after 24 h of infection, the time required for Green Fluorescent Protein (GFP) expression in infected cells. In samples pretreated with HPMC + *β*-CD (Figure 2A,B), Solution 1, an increase in GFP expression can be observed (about 3.5% vs. control, *p* < 0.05), leading to a reduction of about 34% cell viability compared to the infected control (*p* < 0.05), probably due to both pretreatment and infection (Figure 2C). The cells pretreated with high-dose UA + HPMC + *β*-CD, Solution 2, are suffering from pretreatment, but nonetheless are not infected (Figure 2A,B). Indeed, cell viability was reduced compared to the infected control (approximately 56% *p* < 0.05; Figure 2C), but GFP was significantly reduced compared to the control (approximately 9%, *p* < 0.05, Figure 2A,B). Finally, the cells pretreated with Solution 3 and HEXEDRA+ were analyzed. In both cases, cell viability (Figure 2C) was statistically improved (*p* < 0.05) compared to the infected control and compared to Solution 1 and Solution 2 (*p* < 0.05). However, the main effect was particularly evident in the samples with HEXEDRA+, where the addition of Tpmix in the formulation seems necessary for the maintenance of cell viability (approximately 66% vs. Solution 3, *p* < 0.05). Furthermore, the GFP expression (Figure 2A,B) of Solution 3 and HEXEDRA+ revealed the ability to completely block the virus (respectively about 13% and about 22.5% compared to the infected control) as reported by the literature on the UA effect to counteract the coronavirus. In addition, further experiments were needed to evaluate UA content in cells (Figure 2D) to exclude that UA-containing formulations have been absorbed by the cells. The quantification of UA showed that UA present on all formulations tested is not present intracellularly but remains in the extracellular compartment which is represented by the stimulation medium and wash buffer data. These data demonstrate that the UA present in all the formulations tested did not enter the cells, supporting its role on the mechanical barrier function.

### 2.4. Infection with Supernatant Containing VSV-Based Pseudovirus SARS-CoV-2

**Figure 3:**
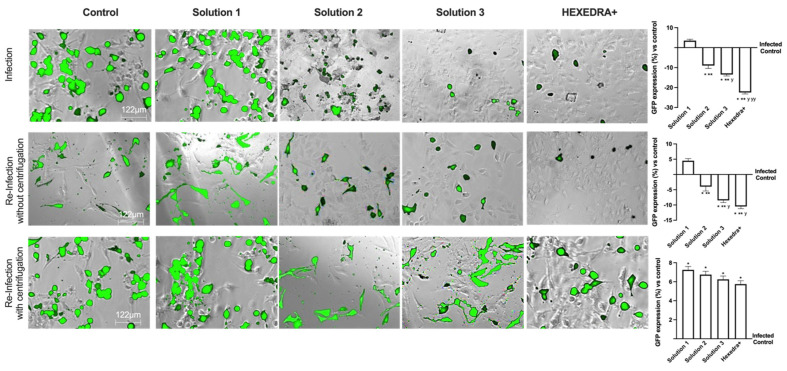
Analysis of infection of Vero E6 cells with VSV-based pseudovirus SARS-CoV-2 (MOI 0.1) pre-treated with the different UA-based formulations. The images reported were an example of each stimulation for GFP fluorescence expression obtained at 40× magnification by Paula (Leica DM1000). The positive cells were counted analyzing GFP expression in cells by the Image J software and the results were expressed as mean ± SD (%) compared to infected control cells (0 line) of four independent experiments. The abbreviations are the same reported in Figure 2. * *p* < 0.05 vs. infected control; ** *p* < 0.05 vs. Solution 1; ^y^ *p* < 0.05 vs. Solution 2; ^yy^ *p* < 0.05 vs. Solution 3.

Based on the previous data, it is important to exclude that in the wells treated with Solution 2, Solution 3, and HEXEDRA+, the infection did not occur due to UA-mediated virus death (excluding direct viral biological function). The supernatants were used as they were (without centrifugation) to infect new Vero E6 cells, i.e., virgin cells lacking any previous stimulation (UA or infection). As reported in Figure 3, the infection picture changes and begins to outline the operational profile of UA. Indeed, the control is found to be infected, as in the previous condition, and the supernatant without centrifugation is able to infect the virgin Vero E6 indicating that a good deal of viral load remains in the supernatant to re-infect other cells. The supernatant of the infection in the presence of Solution 1 demonstrated that it has some viral load able to induce the infection (about 4.5% compared to control of re-infected cells, *p* < 0.05) with a great rate (about 22%) compared to the first infection (*p* < 0.05). On the contrary, for the stimulations with Solution 2, Solution 3, and HEXEDRA+, the supernatants collected after infection do not seem to be able to infect virgin cells. It is impossible to discern whether this is a biological effect of UA against the virus or whether the crosslinker of UA mixed with HPMC retains the virus for a long time. These data were confirmed by the GFP quantification of virgin Vero E6 cells that were not infected in all cases compared to the control (*p* < 0.05).

To verify the hypothesis about the ability of UA + HPMC to trap the virus, the collected supernatants from Vero E6 cells infected after the stimulation with all solutions tested were centrifugated before being added to the virgin Vero E6 cells. As revealed in Figure 3, all cells treated with centrifugated supernatant showed a more significant infection compared to control, as reported by GFP analysis (*p* < 0.05). Indeed, the control is found to be infected, indicating that a good deal of viral load still remains in the supernatant after centrifugation, supporting that this protocol did not affect the virus’ ability to re-infect other cells. The supernatant of *β*-CD +HPMC treatment (Solution 1) improves its viral infection ability (*p* < 0.05 vs. without centrifugation, about 39%), indicating that the viscosity of the formulation contributes to viral adhesion. Finally, concerning Solution 2, Solution 3 and HEXEDRA+, the supernatants collected after infection are found to be able to infect other cells improving their efficiency of infection, probably due to the release of viral particles during centrifugation. In particular, HEXEDRA+ showed a greater GFP expression compared to both Solution 2 (about 40%), Solution 3 (about 20%) and this effect were also significant to the infection without centrifugation (*p* < 0.05, about 60%). All these findings revealed that the different formulations do not appear to have biological activity but rather a predominantly mechanical capacity; they can trap the virus inside without inactivating it and release it after centrifugation.

### 2.5. Infection with Supernatant Containing VSV-Based Pseudovirus SARS-CoV-2 in HNEpC Cells

In the last set of experiments, to exclude any biological interactions between the virus and UA, additional experiments on infection of the virgin population were performed using a different cell type which is mainly involved in virus infection. For this reason, the supernatants of Vero E6 cells after centrifugation were used to infect a HNEpC cell, a cell line phenotypically different from the previous one.

**Figure 4:**
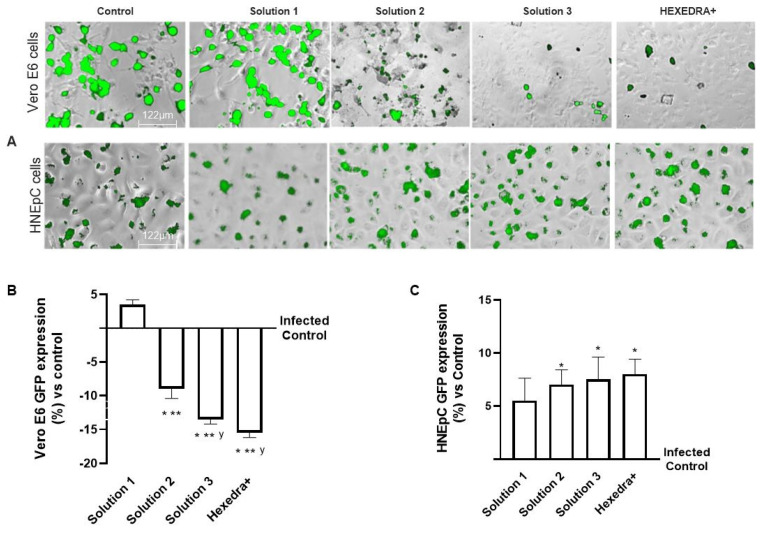
Analysis of infection of Vero E6 and HNEpC cells with VSV-based pseudovirus SARS-CoV-2 (MOI 0.1) pre-treated with the different UA-based formulations. The images reported were an example of each stimulation for GFP fluorescence expression obtained at 40× magnification by Paula (Leica DM1000). The positive cells were counted analyzing GFP expression in cells by the Image J software and the results were expressed as mean ± SD (%) compared to infected control cells (0 line) of four independent experiments. The abbreviations are the same reported in Figure 2. * *p* < 0.05 vs. infected control; ** *p* < 0.05 vs. Solution 1; ^y^ *p* < 0.05 vs. Solution 2.

As can be observed in Figure 4, the post-infection supernatant also induced viral infection in the nasal cells, indicating that UA in the different formulations has no biological activity and is unable to interact directly with the virus. Indeed, the control is infected as before, indicating that in the supernatant, even if centrifuged, remains a good deal of viral load capable of re-infecting other cells of different types. The supernatant from the treatment with *β*-CD +HPMC (Solution 1), as shown in Figure 4, was also able to infect new cells in turn despite the different cell groups used (about 5.5% compared to control). Finally, also for the formulations with Solution 2, Solution 3, and HEXEDRA+, the supernatants collected after infection appear to be able to infect other cells, as can be easily appreciated from the fluorescence analysis. However, between them, the efficacy is similar without statistical changes (*p* < 0.05), indicating that there is no interaction between UA and the virus since the latter was able to infect human nasal cells quite differently from the starting group. Therefore, it can be hypothesized that UA does not appear to alter the specificity of the virus antigenic protein with the specific receptor located on the host cell.

## 3. Discussion

The discovery of new strategies to block nasal cavity infection is critical to the development of preventive and therapeutic strategies, especially since the SARS-COV-2 pandemic broke out. Therefore, the development of a new medical approach as a host-oriented prophylaxis could quickly offer the necessary protection [23]. In this scenario, UA, discovered in 1844 and found mainly in some species of lichens such as *Alectoria, Cladonia, Evernia, Lecanora, Ramalina* and *Usnea* [10], encountered much interest. In nature, it has two different isomers (−) and (+), and a racemic mixture, which is one of the few UA forms commercially available [24]. The main clinical limitation of the human use of UA depends on its physicochemical characteristics and potential hepatotoxicity. These are the main problems that must be solved by applying modifications to the structure of the UA or with suitable formulations capable of improving its biological properties by reducing its collateral effects. For this reason, several approaches, including UA salts or nanoencapsulated formulations, have been shown to improve the bioavailability and safety of UA in animal models, but need to be verified in humans [25]. UA has been widely used as an ingredient in daily personal care products such as toothpastes or mouth rinse. However, it has also been used to promote weight loss, albeit with a limitation of its concentration. Indeed, UA shows hepatotoxicity at very high concentrations, inducing the decoupling of oxidative phosphorylation which leads to damage to mitochondrial respiration [10]. Furthermore, UA exhibits several other beneficial properties such as antibacterial, antiviral, antiprotozoal, and cytotoxic activities [24], as well as other biological properties such as anti-inflammatory, antitumor, and antioxidant activities [12]. In some cases, UA has already been shown to exert antiviral activity against Herpes simplex type 1, Polio type 1, and papilloma virus [12], and recent research has explored its efficacy and safety for the treatment of COVID-19 as well [10].

Despite the numerous reports on the biological activity of UA, knowledge on the specific mechanism of action as a mechanical barrier is very limited and, in this context, structure–activity studies are extremely important as they indicate which characteristics of the compound are crucial for his activity [25].

The COVID-19 pandemic has caused damage in the world by causing health, social, and economic problems. The world needs to improve various therapies and vaccines to gradually return to normal life. Furthermore, effective strategies that can rapidly counter viral spread and help counteract different viral strains must also be cheaper and simpler for all populations. For this reason, through relevant guidelines, several promising strategies are being explored, such as nasal spray, in experimental models in vitro or in vivo [3].

On the basis of the results obtained, this work aims to be proof that the action of UA combined with other elements, such as tocotrienols and HPMC, can create a barrier effect that can trap viruses; indeed, in the literature it is already known that UA has an anti-replicative effect against some viruses [18]. In this experimental context, the barrier effect of UA was already appreciable after an incubation of 3 h at 37 °C. In particular, to the observation under the microscope, the most appreciable visual effect is given by HEXEDRA+, where the presence of branches can be significantly detected. As for the other tested solutions, Solution 2 and Solution 3 are still able to create branches which are not sufficient to create a uniform branching structure. As expected, Solution 1 creates a very weak branched structure, as it contains only the gelling compound. In this regard, it is important to note that in this context the UA cannot exert a biological effect but only a mechanical one; in fact, the supernatant of the pretreated cells, after minimal centrifugation in order to separate viral particles trapped in the UA network (without broken the branch) and to confirm the absence of HEXEDRA+ biological activity, was still able to infect the virgin cells, thus demonstrating that the virus maintained its infectious power. It can be observed that the virgin cells of the control suffer fewer infections because, in the previous experiment, most of the virus had already entered the cells. Therefore, the number of viral particles that remain inside the supernatant is insufficient to infect 10,000 cells since the MOI is less than 0.1. A similar behavior is observed in cells pretreated with Solution 1, since the barrier effect is not appreciable due to the solution consisting only of HPMC.

Conversely, the supernatant of cells pretreated with Solution 2, Solution 3, and HEXEDRA+ can infect virgin cells because the barrier effect exerted by the UA could hinder the entry of the virus into the cells by masking the specific receptor. Indeed, the virus remained trapped in the film formed by UA + HPMC and when the film was removed, the cells, which were washed, remained unacted on without the presence of UA. For this reason, it was possible to deduce that the viral activity remained intact without any alteration, as evidenced by the expression of GFP, which was visible after infection. Furthermore, infection with Vero E6 supernatant on HNEpC cells did not appear to be altered, thus ruling out the possibility of a possible virus–cell-specific receptor interaction. In summary, this work has shown that UA could trap viral particles and, more importantly, this ability is due to a mechanical rather than a biological effect.

This preliminary study suggests that HEXEDRA+ could be a safe, non-drug and easy-to-use nasal spray that could reduce the risk of infection by creating a mechanical barrier against SARS-CoV-2 and potentially all airborne viruses. It appears to be a promising self-protection strategy to be used as a complement without interfering with the existing preventive health measures. However, this very promising nasal spray requires further study before its clinical application.

## 4. Materials and Methods

### 4.1. Agents Preparation

The solutions prepared for the experiments were composed as follows: 150 mcg/mL *β*-CD + 1% HPMC (named Solution 1); 150 mcg/mL UA+ 150 mcg/mL *β*-CD + 1% HPMC (named Solution 2); 75 mcg/mL UA + 150 mcg/mL *β*-CD +1% HPMC (named Solution 3); 75 mcg/mL UA+ 150 mcg/mL *β*-CD +0.1% TPmix +1% HPMC (named HEDEXRA+). The final combination was prepared according to the PCT/IB2021/062486 patent (Vestatis GMBH, Gruner Deich, 1-3, 20,097 Hamburg (DE)) and was the same used to prepare a commercial product for human use in the form of a nasal spray, classified as a Class II Medical Device. The selected form of usnic acid is (+)- Usnic acid with a molecular rotation of about 500°, extracted from natural source. The purity of this molecule ranges from 97% to 98%, and the ideal granule size is between 10 and 20 microns. In addition, the particle distribution range is preferably between 0.2 and 15µm. The polymer selected, HPMC, was used in an amount of preferably 1% which is necessary to create an aqueous gel. All formulations were tested to evaluate by which mechanism (mechanical barrier or biological interaction) the UA acts when it comes into contact with a biological sample and a viral contaminant. The concentrations of UA used were identified in a previous experiments reported in the patent.

### 4.2. Staining Membrane

Since UA is a natural extract derived from lichen, it is possible to hypothesize a targeted staining aimed at identifying structural components by exploiting a technique used for compounds derived from animal source, such as using a classical staining with hematoxylin and eosin (Merck Life Science, Rome, Italy) [26] with few modifications to assess the morphological traits of UA reticulates. In particular, UA samples were diluted 1:1 with hematoxylin and then deposited on an empty cell-free plate and placed in incubator for 3 h. At the end, the supernatants were removed and after washing with water, the bottom of each well was observed under a light microscope (Leica DM1000).

### 4.3. Cell Culture

Vero E6 cells donated from the Laboratory of Clinical Medicine Dept. of Translational Medicine, University of Eastern Piedmont (UPO), were grown as described in the literature [27] maintaining cells in Dulbecco’s modified Eagle’s medium (DMEM; Thermo Fisher, Milan, Italy) supplemented with 10% foetal bovine serum (FBS; Merck Life Science, Rome, Italy), 0.075% Sodium Bicarbonate (7.5% solution, Thermo Fisher, Milan, Italy) and 1x Pen-strep (Thermo Fisher, Milan, Italy) and kept under 5% CO_2_ at 37 °C. The cells were plated 1 × 10^4^/well in a 96-well multiplate to analyze the cell viability and infection state and 1 × 10^5^/well in a 24-well multiplate to quantify UA on both supernatants and cell lysates.

HNEpC, purchased from PromoCell (Heidelberg, Germany), were utilized in a widely used model to study the pathogenicity of several viruses in the human airway epithelium in which both ACE2 and TMPRSS2 receptors are expressed [28]. Cells were cultured using a commercially available airway epithelial cell growth medium with supplements (Airway Epithelial Cell Growth Medium Ready-to-use, PromoCell, Heidelberg, Germany) at 37 °C, 5% CO_2_. The cells were plated 1 × 10^4^/well in a 96-well multiplate to analyze the cell viability and infection state and 1 × 10^5^/well in a 24-well multiplate to quantify UA on both supernatants and cell lysates. For both cell types, the assay medium contained 2% FBS and pseudovirus SARS-CoV-2 [29]. In addition, both cell types were used at low passages and only after verification by PCR of conservation of ACE2 and TMPRSS2 receptors. Confirmation of the presence of these receptors is important to determine that the model is consistent with the study of SARS-CoV-2 pathogenesis [30].

### 4.4. Experimental Protocol

The experiments were divided into three steps in order to investigate the ability of nasal spray composed of UA + HPMC+ *β*-CD -TPmix to create and maintain a mechanical barrier to prevent the entry of the virus such as VSV-based pseudovirus SARS-CoV-2. In the first set of experiments, the tested substances were administered as pretreatment for 3 h on Vero E6 cells, then the medium was removed, the cells were washed with sterile PBS 1X buffer and then infected with VSV-based pseudovirus SARS-CoV-2 for 3 h.

In the second set of experiments, the supernatant of Vero E6 cells at the end of the 3 h infection with VSV-based pseudovirus was collected and used either as it is or centrifuged at 200 rpm for 2 min on a blank population (never previously treated) of Vero E6 cells. The centrifugation procedure was performed with the aim of separating the viral particles from the AU-generated network.

In the third set of experiments, the supernatant of Vero E6 cells at the end of the 3 h infection with VSV-based pseudovirus was collected and centrifuged at 200 rpm for 2 min and used on a different blank population (untreated) of HNEpC cells.

This assay was essential to determine both the barrier effect and to be able to verify the unchanged capacity for VSV-based pseudovirus SARS-CoV-2 to infect the cells.

Infected cells were photographed with a fluorescence microscope (Paula, Leica Microsystems, Wetzlar, Germania) and compared with each other as well as with the control sample.

At the same time in the first and in the last set of experiments, cell viability and UA dosage were also performed to demonstrate the safety of the combination tested.

### 4.5. PCR Analysis

RT-PCR was performed as reported in the literature [31]. For both cell types, 7.5 × 10^4^ cells were seeded and left to grow. After 24 h the medium was aspirated and 500 µL of nucleosol (MACHEREY-NAGEL GmbH & Co. KG) was added to the cells. Then 200 µL of RNAse- and DNAse-free water (Invitrogen, Milan, Italy) were mixed with the sample and centrifuged at 14,000 rpm at 4 °C for 15 min. An amount of 600 µL of isopropanol was added to 600 µL of supernatant and incubated for 5 min at room temperature. The sample was centrifuged for an additional 15 min at 14,000 rpm at 4 °C to pellet the RNA. Finally, RNA was resuspended in 50 µL of ultrapure water and then 2 µL of RNA material were quantified using NANODrop2000 (ThermoFisher, Milan, Italy) at 260 and 280 nm. Approximately 1 µg of total RNA was used for cDNA synthesis using the Maxima first-strand cDNA synthesis kit (ThermoFisher, Milan, Italy). The tube reaction had a final volume of 20 µL (10 µL of RNA sample and 10 µL of reaction mix) and a 2.5 h retrotranscription program was started, which involves a priming step at 25 °C for 10 min, a pairing step at 37 °C for 2 h, and finally a denaturation step at 85 °C for 5 min. Afterwards, the samples were suspended in 8 µL of ultrapure water. Real-time qPCR reactions were performed on a CFX96 (Bio-Rad Laboratories, Hercules, CA, USA) apparatus with 5x Powertrack syber green master mix (Kit High Capacity cDNA Reverse Transcription Kit, Applied Biosystems, Milan, Italy). Each sample was prepared in duplicate. To reduce systematic errors, 18 µL of Specific Mix was placed in the first well to which 2 µL of cDNA was added, and after mixing the contents, 10 µL of the total 20 were obtained and moved to the next well. The program in the thermal cycler (Bio-Rad Laboratories, Hercules, CA, USA) was then started, which involves a cycle at 95 °C for 2 min to activate the enzyme, followed by 40 cycles in which denaturation is alternated at a temperature of 95 °C for 5 s and the pairing and extension phase operate at 60 °C for 30 s.

The following primers were used for relative gene expression quantification (Table 3):

### 4.6. Cell Viability

Both cell types at the end of infection were incubated with 1 mg/mL 3-(4,5-Dimethylthiazol-2-yl)-2,5-diphenyltetrazolium bromide (MTT dye) (MTT-based in vitro Toxicology Assay Kit, Merck Life Science, Rome, Italy) in DMEM without both phenol red and FBS (Merck Life Science, Rome, Italy) for 2 h at 37 °C in an incubator to analyze cell viability measuring the absorbance at 570 nm with a correction at 690 nm by a spectrometer (Infinite 200 Pro MPlex, Tecan, Milan, Italy). The results were reported as means ± SD (%) to control cells (baseline 0 line) [32].

### 4.7. Pseudovirus Infection

Vero E6 cells were pretreated with the different selected compounds for 3 h before proceeding with infection using VSV-based pseudovirus SARS-CoV-2 (DBA Italia S.r.l., Milan, Italy) as described in the literature [33]. Briefly, the viral vial was used at MOI 0.1 in a medium incubating cell for 3 h at 37 °C, then the medium was changed adding complete culture medium to maintain cells in the incubator for 24 h. All samples and viruses were diluted with 2% FBS-DMEM. After 24 h of incubation, fluorescence images were obtained with Paula (Leica Microsystems, Wetzlar, Germania). For quantitative determination, fluorescence images were analyzed by ImageJ software, and the numbers of GFP-positive cells (expression of virus-associated fluorescence) for each well were counted to represent infection performance [34]. Fluorescent cells were counted, and the data obtained were evaluated with statistical program by comparing the data with positive control values (considered 100% infection).

### 4.8. UA Dosage

UA presence was performed as reported in the literature [35], analyzing the empty plate, each specific cell lysate, and the supernatant and wash buffer. Briefly, each supernatants post infection and cell monolayer was collected; in particular, cell monolayer was lysed in cold PBS1x with a cell scraper, mixed for 30 min at 4 °C, and centrifuged for 20 min at 13,000 rpm at 4 °C. At the end of centrifugation, the supernatants from lysate and from cell culture were obtained and analyzed by spectrophotometer (Infinite 200 Pro MPlex, Tecan, Milan, Italy) at 281 nm. The results were expressed as ng/mL.

### 4.9. Statistical Analysis

Data reported were obtained from at least four independent experiments performed in triplicate for each experimental protocol and analyzed using Prism GraphPad statistical software. Results are reported as means ± SD (standard deviation) using one-way ANOVA followed by the Bonferroni post hoc test for statistical analysis. Values of *p* < 0.05 were considered statistically significant.

## 5. Conclusions

As demonstrated for the first time from the results presented in this study, HEXEDRA+ can form a mechanical barrier capable of blocking viral infection by means of an entrapment mechanism due to the specific properties of the combination, confirming the importance of the structure of UA and its use in combination with other substances. Furthermore, these data showed that UA did not exert a direct biological interaction with the virus; it is also evident that a virus–cell-specific receptor interaction was also excluded. Furthermore, these data showed that UA can be used safely by modifying the route of administration and maintaining its positive effects. In any case, although these results are very promising, before testing this medical device on humans, it is necessary to proceed with further experiments to exclude adverse effects both in in vitro and in vivo studies.

## Figures and Tables

**Table 1:** UA spectrophotometry quantification at 281 nm by Tecan (Infinite 200 Pro MPlex, Tecan, Milan, Italy) and data are expressed as µg/mL ± SD of four independent experiments. Solution 1 = 150 mcg/mL *β*-CD + HPMC; Solution 2 = 150 mcg/mL UA + 150 mcg/mL *β*-CD + 1% HPMC; Solution 3 = 75 mcg/mL UA + 150 mcg/mL *β*-CD + 1% HPMC; HEXEDRA+ = 75 mcg/mL UA + 150 mcg/mL *β*-CD + 0.1% Tpmix + 1% HPMC.

	Solution 1	Solution 2	Solution 3	HEXEDRA+
Wash buffer	0 µg/mL	2.1 ± 0.5 µg/mL	1.95 ± 0.8 µg/mL	1.97 ± 0.9 µg/mL
Bottom wash	0 µg/mL	1.9 ± 1.7 µg/mL	1.88 ± 1.3 µg/mL	1.88 ± 0.8 µg/mL

**Table 2:** Values obtained from 4 independent experiments conducted in triplicate and expressed as mean ± SD on both cell populations are shown. Hypoxanthine phosphoribosyltransferase 1 (HPRT) was used as an internal PCR control. Assays were conducted before each experiment to ensure that subsequent infection could occur, thus excluding cell culture-related confounders.

Receptor	Cell Typology	PCR Cycle (Mean ± SD)
ACE2	Vero E6	25.71 ± 1.01
	HNEpC	30.7 ± 1.35
TMPRSS2	Vero E6	34.27 ± 1.07
	HNEpC	29.43 ± 0.10
HPRT	Vero E6	19.59 ± 0.51
	HNEpC	23.20 ± 0.44

**Table 3:** Primers used to verify the correct cell morphology receptors.

Gene	Sense	Sequence
HPRT	Forward	5′-GATTTGGAAAGGGTGTTTAT-3′
	Reverse	5′-TCCCATCTCCTTCATCACAT-3′
ACE 2	Forward	5′-TCCATTGGTCTTCTGTCACCCG-3′
	Reverse	5′-AGACCATCCACCTCCACTTCTC-3′
TMPRSS2	Forward	5′-CCTCTAACTGGTGTGATGGCGT-3′
	Reverse	5′-TGCCAGGACTTCCTCTGAGATG-3′

## Data Availability

Raw data are preferably deposited at the Laboratory of Physiology (F.Uberti), ensuring appropriate measures so that raw data are retained in full forever under a secure system. The data presented in this study are available on reasonable request from the corresponding author.

## Abbreviations

ACE2	angiotensin-converting enzyme 2
DMEM	Dulbecco’s modified Eagle’s medium
FBS	foetal bovine serum
FDA	US Food and Drug Administration
GFP	Green Fluorescent Protein
HNEpC	human nasal primary cells
HPMC	hydroxypropyl methylcellulose
HPRT	hypoxanthine phosphoribosyltransferase 1
MOI	multiplicity of infection
MTT	3-(4,5-Dimethylthiazol-2-yl)-2,5-diphenyltetrazolium bromide
PBS	phosphate buffered saline
ROS	radical oxygen species
SD	standard deviation
TMPRSS2	transmembrane serine protease 2
TPmix	tocopherol and tocotrienol
UA	usnic acid
Vero E6	African green monkey kidney cell line
VSV	recombinant vesicular stomatitis virus
*β*-CD	2-hydroxypropyl-beta-cyclodextrin
